# Metagenomic versus targeted next-generation sequencing for detection of microorganisms in bronchoalveolar lavage fluid among renal transplantation recipients

**DOI:** 10.3389/fimmu.2024.1443057

**Published:** 2024-08-26

**Authors:** Zhaoru Huang, Bingxue Hu, Jinfeng Li, Min Feng, Zhigang Wang, Fengxiang Huang, Huan Xu, Lei Liu, Wenjun Shang

**Affiliations:** ^1^ Kidney Transplantation Department, the First Affiliated Hospital of Zhengzhou University, Zhengzhou, China; ^2^ Center for Infectious Diseases, Vision Medicals Co., Ltd, Guangzhou, China; ^3^ Surgical Intensive Care Unit, the First Affiliated Hospital of Zhengzhou University, Zhengzhou, China; ^4^ Respiratory Department, the First Affiliated Hospital of Zhengzhou University, Zhengzhou, China

**Keywords:** kidney transplantation, pulmonary infection, metagenomics Next-Generation Sequencing (mNGS), targeted next-generation sequencing (tNGS), respiratory pathogens, Torque teno virus

## Abstract

**Background:**

Metagenomic next-generation sequencing (mNGS), which provides untargeted and unbiased pathogens detection, has been extensively applied to improve diagnosis of pulmonary infection. This study aimed to compare the clinical performance between mNGS and targeted NGS (tNGS) for microbial detection and identification in bronchoalveolar lavage fluid (BALF) from kidney transplantation recipients (KTRs).

**Methods:**

BALF samples with microbiological results from mNGS and conventional microbiological test (CMT) were included. For tNGS, samples were extracted, amplified by polymerase chain reaction with pathogen-specific primers, and sequenced on an Illumina Nextseq.

**Results:**

A total of 99 BALF from 99 KTRs, among which 93 were diagnosed as pulmonary infection, were analyzed. Compared with CMT, both mNGS and tNGS showed higher positive rate and sensitivity (p<0.001) for overall, bacterial and fungal detection. Although the positive rate for mNGS and tNGS was comparable, mNGS significantly outperformed tNGS in sensitivity (100% vs. 93.55%, p<0.05), particularly for bacteria and virus (p<0.001). Moreover, the true positive rate for detected microbes of mNGS was superior over that of tNGS (73.97% vs. 63.15%, p<0.05), and the difference was also significant when specific for bacteria (94.59% vs. 64.81%, p<0.001) and fungi (93.85% vs. 72.58%, p<0.01). Additionally, we found that, unlike most microbes such as SARS-CoV-2, *Aspergillus*, and EBV, which were predominantly detected from recipients who underwent surgery over 3 years, Torque teno virus (TTV) were principally detected from recipients within 1-year post-transplant, and as post-transplantation time increased, the percentage of TTV positivity declined.

**Conclusion:**

Although tNGS was inferior to mNGS owing to lower sensitivity and true positive rate in identifying respiratory pathogens among KTRs, both considerably outperformed CMT.

## 1 Introduction

Renal transplantation has emerged as a crucial therapeutic intervention for individuals with end-stage chronic kidney disease. However, common postoperative complications include secondary infection, acute rejection, chronic rejection, and delayed graft function, and other similar issues may occur. Among them, the pathogens and symptoms of secondary infection vary greatly, which can result in the loss of graft function and even the death of the recipient, thus has garnered significant attention (1). It was reported that the overall hospital admission rates of renal transplants due to infections were approximately 35% (2–4). The postoperative infection among kidney transplantation recipients (KTRs) following transplantation frequently affects the respiratory system, with the majority of cases being characterized by mixed infections. If the appropriate intervention measures are not implemented promptly after the onset of pulmonary infection, it frequently advances swiftly to acute respiratory distress syndrome (ARDS), which can bring about a life-threatening risk to severely affected individuals (5). Identifying the specific causative microorganisms, administering antibiotics with precision, and modifying the usage of immunosuppressive medications comprise the main approaches to treating pulmonary infection among KTRs (1).

Following transplantation, the diagnosis of infection is typically made using a combination of clinical symptoms and laboratory diagnostic techniques, such as histopathology, culture of various bodily fluids and tissues, serology detection, microscopic cell detection, and nucleic acid detection (5). Nevertheless, these conventional microbiological tests (CMTs) are time consuming and may result in false negative results (6). Conversely, metagenomic next-generation sequencing (mNGS), as a culture-independent method, enables the fast and precise sequence detection of all microorganisms (such as bacteria, fungus, viruses, and parasites) in a single clinical sample without bias (7, 8). mNGS has been successfully used in organ transplantation, such as hematopoietic stem cell and liver, lung, and kidney transplantations (9–13). As for the application of mNGS in diagnosing pulmonary infection among KTRs, studies mainly focused on *Pneumocystis jirovecii* pneumonia (PJP) (14–17), and no research has been performed to comprehensively evaluate the performance of mNGS in identifying respiratory pathogens.

Targeted next-generation sequencing (tNGS) is a more affordable method that covers overwhelming majority of respiratory pathogens by enriching species-specific sequences. The panel of the tNGS was ranged from dozens to hundreds of pathogens based on the designed primes (18–20). Thus, tNGS only can detect the pathogens in the panel. However, mNGS has the potential to detect all the pathogens including rare and newly occurred pathogens. Whether these trade-offs have a discernible effect on the outcomes is still unknown. Until now, few studies were reported to compare the clinical performance of mNGS and tNGS in the diagnosis of infectious disease. Herein, we sought to evaluate the performance of the mNGS assay and a complementary tNGS assay by taking clinical diagnosis of each bronchoalveolar lavage fluid (BALF) sample as standard. In addition, the correlations between detected microbes and clinical parameters were investigated.

## 2 Materials and methods

### 2.1 Patients and samples

We retrospectively screened medical records of 155 bronchoalveolar lavage fluid (BALF) samples with suspected or diagnosed pulmonary infections who were admitted to the Kidney Transplantation Department of the First Affiliated Hospital of Zhengzhou University in Zhengzhou, China, between January 2022 and September 2023. Samples those sent for both mNGS and CMT (culture, smear, PCR, serum tests, G/GM) within 3 days were reviewed, and were finally included for analysis if they met the following criteria: (i) from patients aged more than 18 years; (ii) from kidney transplantation recipients (KTRs); and (iii) from patients exhibiting common symptoms of pulmonary infection include fever, cough, phlegm, shortness of breath, chest tightness, dyspnea, etc., and hospitalized for more than 24 h. Samples from KTRs combined with other transplants, patients without sufficient information in the electronic medical records, those who died within 24 h after admission, or the finally diagnosed causative pathogens were indefinite were excluded. Final clinical diagnoses for patients were retrospectively determined by an expert panel made up of doctors from the departments of kidney transplantation, respiratory medicine, and microbiologists based on the patients’ characteristics and the composite diagnostic results.

This study was conducted in accordance with the Declaration of Helsinki. Study protocols were reviewed and approved by the Ethics Committee of the First Affiliated Hospital of Zhengzhou University (approval number 2024-KY-0454-001).

### 2.2 mNGS

#### 2.2.1 Host depletion, DNA extraction, library construction, capture hybridization, and sequencing

Briefly, pathogens and human cells were separated from 1-mL samples by centrifuging it at 12,000 *g* for 5 min. The host nucleic acid was then removed from the precipitate using 1 U Benzonase (Sigma) and 0.5% Tween 20 (Sigma), which were incubated at 37°C for 5 min. The nucleic acid was then extracted and eluted from 400 µL of pretreatment samples using a QIAamp UCP Pathogen Mini Kit in 60 µL elution buffer (catalog number 50214, Qiagen, Hilden, Germany). Using a Qubit dsDNA HS Assay Kit (catalog number Q32854, Invitrogen, Carlsbad, CA, USA), the isolated DNA was quantified (21, 22). Total RNA was extracted using QIAamp UCP pathogen minikit (Qiagen, Valencia, CA, USA) before being subjected to human rRNA depletion (Vazyme, Nanjing, China). For the creation of cDNA, 10 µl of purified RNA was employed. The KAPA low throughput library construction kit (KAPA Biosystems, Boston, MA, USA) was used to create a DNA/cDNA library in accordance with the manufacturer’s instructions^2^. An aliquot of 750-ng library from each sample was used for hybrid capture-based enrichment of microbial probe one rounds of hybridization (SeqCap EZ Library, Roche, Pleasanton, CA, USA). Probes were designed using CATCH pipeline (23). A Qubit dsDNA HS assay kit was used to measure the library concentration. Library quality was assessed with an Agilent 2100 Bioanalyzer (Agilent Technologies, Santa Clara, CA, USA) using a high-sensitivity DNA kit. The library was prepared by pooling a 1.5-pM concentration of each purified sample equally for sequencing on an Illumina NextSeq 550 sequencer using a 75-cycle single-end sequencing strategy.

#### 2.2.2 Bioinformatic analysis

Trimmomatic was used to eliminate low-quality reads, duplicate reads, adapter contamination, and those shorter than 70 bp (24). Low-complexity reads were removed by Kcomplexity’s default settings. By utilizing SNAP v1.0beta.18 to match the human sequence data to the hg38 reference genome, the human sequence data were located and eliminated (25). The Kraken 2 criteria for choosing representative assemblies for microorganisms (bacteria, viruses, fungi, protozoa, and other multicellular eukaryotic pathogens) from the NCBI Assembly and Genome databases (https://benlangmead.github.io/aws-indexes/k2) were used to select pathogens and their genomes or assemblies for the creation of the microbial genome database. Microbial reads were aligned to the database using Burrows–Wheeler Aligner software (26). The reads with 90% identity of reference were defined as mapped reads. In addition, reads with multiple locus alignments within the same genus were excluded from the secondary analysis. Only reads mapped to the genome within the same species were considered.

To remove the mistakes brought on by different sequencing depths between samples, we normalized the sequencing reads using RPTM. Samples spiked with microorganisms were classified as positive samples, and NC was defined as the negative sample. The parameter resulting in the highest area AUC was considered the positive cutoff value for this species (27). For microorganisms without culture isolates, the RPTM mean value and standard deviation of this microorganism were calculated, and the RPTM (mean + 2SD) was set as a positive cutoff value (28).

The clinical reportable range (CRR) for pathogens was established according to the following three references indicated in a previous study (28): I. Johns Hopkins ABX Guide, II. Manual of Clinical Microbiology (29), and III. clinical case reports or research articles published in peer-reviewed journals.

### 2.3 tNGS

tNGS-targeted microbial species in this study are listed in **Supplementary Table S1**. DNA extraction was performed using a whole DNA extraction kit (Guangdong Sui Equipment Preparation no. 20191662) that was independently produced by Vision Medicals Co., Ltd. (Guangzhou, China). Using an RNA extraction kit (no. 20201360), total RNA was extracted. Reverse transcription into cDNA was then carried out using reverse transcriptase and dNTPs (Thermo Fisher, USA). Multiple PCR was used to amplify the extracted DNA and cDNA using a pathogen-specific primer mix (30, 31), with a reaction cycle as follows: pre-denaturation at 95°C for 3 min, denatured at 95°C for 20 s, and annealed at 60°C for 4 min, a total of 25 cycles were run, with the reaction terminating at 16°C after the cycle was stopped and extended for 4 min at 72°C. Then, a barcode primer was used for the second round of PCR amplification, which was conducted using the first round’s product as a template (pre-denaturation at 95°C for 3 min, denatured at 95°C for 15 s, annealed at 58°C for 15 s, and extended at 72°C for 1 min). After seven cycles, the cycle was extended for 10 min at 72°C, and the reaction was terminated at 10°C. The concentration of the generated library was assessed using the Qubit 4.0 nucleic acid fluorometric assay and its corresponding Qubit dsDNA HS Assay kit (Thermo Fisher, USA) upon purification. For 50 single-ended sequencing cycles, the library was put onto the Illumina Nextseq CN500 sequencer.

Trimmomatic software was used to eliminate low-quality sequences, sequences shorter than 40 bps, and junction sequences from the sequencing data to obtain high-quality data. The following bioinformatic analysis and data interpretation processes were consistent with mNGS.

### 2.4 Definition of sensitivity, specificity, true positive, false positive, true negative, and false negative

As reference to clinical diagnosis, true positive (TP) was defined as positive detection in clinical diagnosis positive, false positive (FP) was defined as positive detection in clinical diagnosis negative, true negative (TN) referred to negative detection in clinical diagnosis negative, and false negative (FN) referred to negative detection in clinical diagnosis positive. The sensitivity (TP rate) was defined as [TP/(TP+FN)], while specificity (TN rate) was defined as [TN/(TN+FP)].

### 2.5 Statistical analysis

Continuous variables were expressed as medians [first quartile (Q1), third quartile (Q3)], and non-continuous variables were presented as mean ± standard error (SD). Categorical variables were in counts and percentages unless otherwise specified. The chi-square test was used to compare differences in categorical variables, and the Mann–Whitney U test was employed for continuous variables. Data analysis was performed using GraphPad Prism 6.0 (GraphPad software). Statistical significance was considered to be present when p<0.05.

## 3 Results

### 3.1 Patients’ characteristics

**Figure 1:**
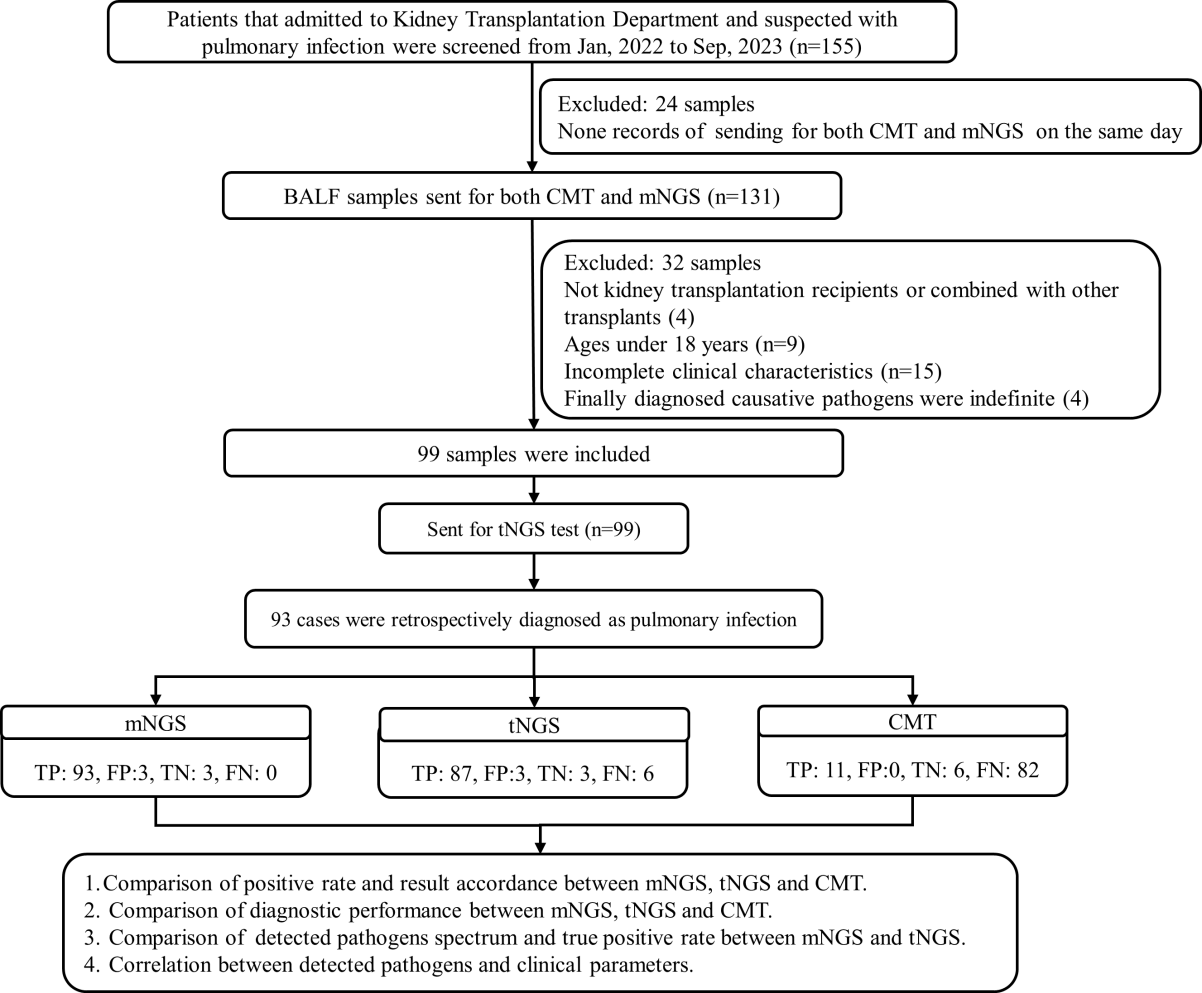
Flowchart of the study.

A total of 99 BALF samples from 99 KTRs were finally investigated in this study (**Figure 1**). The demographic and clinical information of all cases were collected, including sex, age, underlying diseases, dialysis form and durations, post-transplantation time, application of immunosuppressant, and laboratory examinations. As summarized in **Table 1**, there were 67 male and 32 female KTRs with an average age of 39.6 ± 9.3. Before surgery, a majority of patients received hemodialysis (n=80, 80.81%), followed by peritoneal dialysis in 11 patients, and 48 cases (48.48%) underwent dialysis for <1 year (**Table 1**). A total of 56 patients (56.57%) were more than 3 years after kidney transplantation at the time point of this admission, followed by 26 cases who were <1-year post-transplantation, 10 were between 1 years and 2 years post-transplantation, and the rest of the seven KTRs were in 2–3 years post-transplantation. All cases received induction with Glucocorticoid + Anti-human T Lymphocyte Rabbit Immunoglobulin (ATG-F)/Rabbit Anti-human Thymocyte Immunoglobulin (ATG) to prevent renal allograft rejection before surgery. For body weight ≤65kg, intraoperative administration of 50 mg ATG-F/12.5mg ATG was given; for body weight >65 kg, 100 mg ATG-F/25 mg ATG was given. Postoperative treatment was given with 50 mg/day in a total course of 5–6 days. Methylprednisolone 15 mg/kg was given intraoperatively, 7.5 mg/kg on the first day, 5 mg/kg on the second day, 2.5 mg/kg on the third day, 1.25 mg/kg on the fourth day, and 40 mg on the fifth day after surgery, followed by oral prednisone of 20 mg or methylprednisolone 16 mg daily. After the transplant, the maintenance therapy consisted of Tacrolimus or Cyclosporin in combination with Mycophenolate mofetil and Prednisone. The initial concentration for Tacrolimus was set between 0.05 mg/kg.d and 0.25 mg/kg.d, and for Cyclosporin, it was set between 6 mg/kg.d and 8mg/kg.d. The target concentration of Tacrolimus was 10–15 ng/ml within 30 days after transplant, 8–15 ng/ml within 30–90 days, 5–12 ng/ml within 3–12 months, and 5–10 ng/ml over 1 year. The target concentration for Cyclosporin was 200–350 ng/ml within 30 days, 150–300 ng/ml within 30–90 days, 100–250 ng/ml within 3–12 months, and 50–150 ng/ml over 1 year (**Table 2**).

**Table 1:** Characteristics of included patients.

Characteristics	N=99
Sex	
Male	67 (67.68)
Female	32 (32.32)
**Age, years (mean ± SD)**	39.6 ± 9.3
Body weight (kg)	
≤65	63 (63.64)
>65	36 (36.36)
Underlying diseases	
Hypertension	76 (76.77)
Diabetes	4 (4.04)
Heart disease	3 (3.03)
Pre-transplant dialysis form	
Hemodialysis	80 (80.81)
Peritoneal dialysis	11 (11.11)
Hemodialysis+peritoneal dialysis	6 (6.06)
Unknown	2 (2.02)
Pre-transplant dialysis durations	
<1 year	48 (48.48)
1–2 years	17 (17.17)
>2 years	17 (17.17)
Unknown	17 (17.17)
Post-transplantation time	
<1 year	26 (26.26)
1–2 years	10 (10.1)
2–3 years	7 (7.07)
>3 years	56 (56.57)

**Table 2:** Immunomodulator regimen.

Induction agent	
Before surgery	
Body weight (kg)≤65	Glucocorticoid+50 mgATG-F/12.5 mgATG
Body weight (kg)>65	Glucocorticoid+100 mgATG-F/25 mgATG
Intraoperatively	15mg/kg methylprednisolone
Postoperative treatment	
ATG-F/ATG	50 mg/12.5 mg for 5–6 days
Methylprednisolone	
the first day	7.5 mg/kg
the second day	5 mg/kg
the third day	2.5 mg/kg
the fourth day	1.25 mg/kg
the fifth day	40 mg
Maintenance therapy	
Tacrolimus/Cyclosporin+ Mycophenolate mofetil+20 mg prednisone	
The initial concentration of Tacrolimus	
within 30 days	10–15 ng/ml
30–90 days	8–15 ng/ml
3–12 months	5–12 ng/ml
over l year	5–10 ng/ml
The initial concentration of Cyclosporin	
within 30 days	200–350 ng/ml
30–90 days	150–300 ng/ml
3–12 months	100–250 ng/ml
over l year	50–150 ng/ml

### 3.2 Comparison of diagnostic performance for pulmonary infection

**Figure 2:**
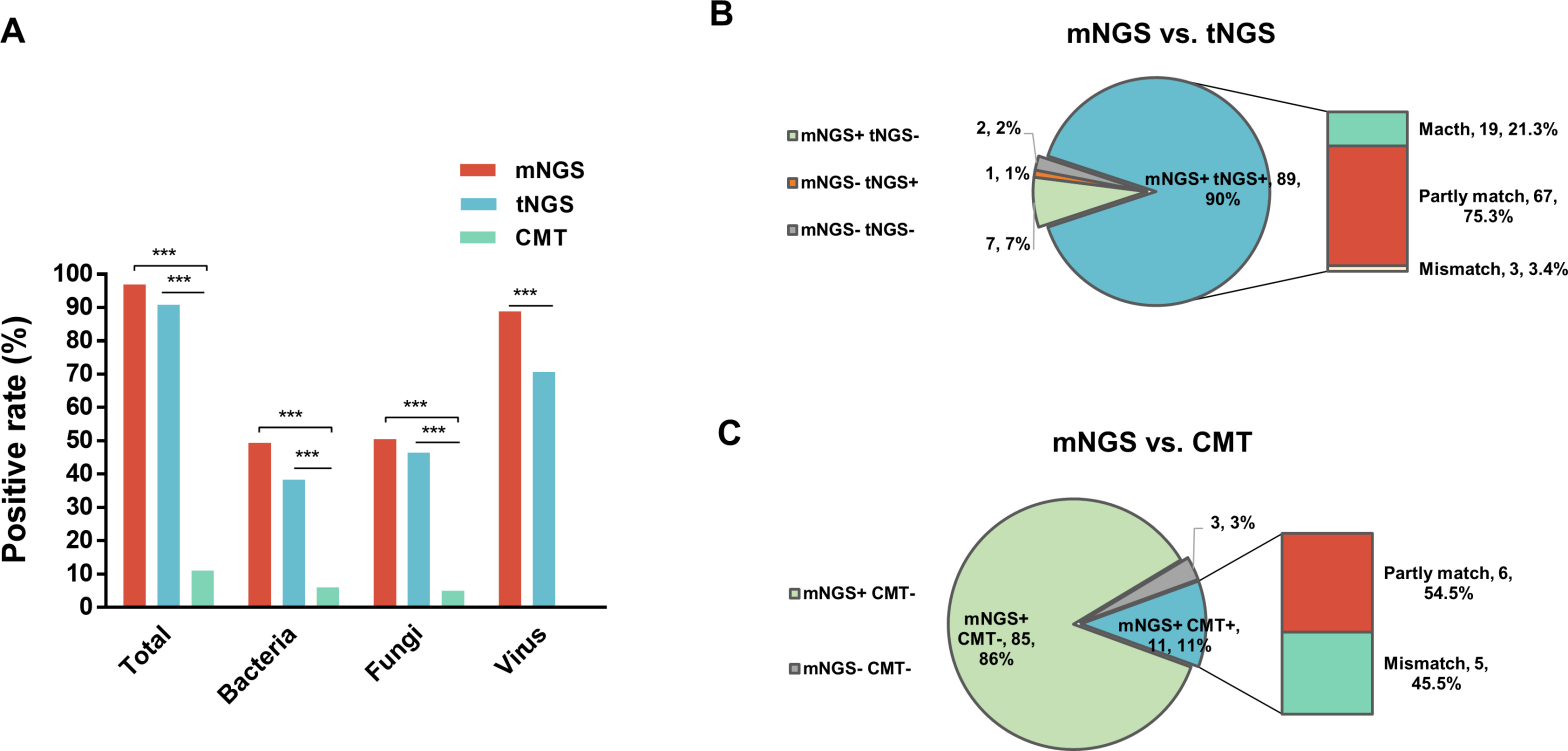
Comparison of positive rate and result accordance. **(A)** Positive rates comparison for total, bacteria, fungi, and virus between mNGS, tNGS, and CMT. **(B)** Pie chart demonstrating the result consistency of mNGS and tNGS, and the double positive results were further categorized as match, mismatch, and partly match. **(C)** Pie chart demonstrating the result consistency of mNGS and CMT, and the double positive results were further categorized as mismatch, and partly match. mNGS, metagenomic next-generation sequencing; tNGS, targeted next-generation sequencing; CMT, conventional microbiological tests. ***P<0.001.

The overall microbial positive rates of mNGS and tNGS were 96.97% (96/99) and 90.97% (90/99), respectively, both significantly higher than that for CMT of 11.11% (11/99, p<0.001). In terms of detection rate for bacteria and fungi, a significantly higher positive rate was also observed for mNGS and tNGS than CMT (49.49% for mNGS bacteria vs. 6.06% for CMT bacteria; 38.38% for tNGS bacteria vs. 6.06% for CMT bacteria; 50.51% for mNGS fungi vs. 5.05% for CMT fungi; and 46.46% for tNGS fungi vs. 5.05% for CMT fungi; p<0.001) (**Figure 2A**). CMT did not detect virus, and mNGS detected a remarkably larger rate of virus than tNGS (88.89% vs. 70.71%, p<0.001). In addition, no significant difference in total positive rate and bacterial and fungal detection rate between mNGS and tNGS was observed (p>0.05) (**Figure 2A**).

As for results consistency of the comparison between mNGS, tNGS, and CMT (**Figures 2B, C**), mNGS and tNGS showed both positive result in 89 of 99 samples (90%) and were both negative in 7 of 99 (7%) samples. Two samples were detected to be positive in mNGS assay only, and one sample was positive in tNGS test only. For 89 double-positive samples, the results of mNGS and tNGS completely matched (positive pathogens were identical) in 19 (21.3%) samples, partly matched (shared at least one positive pathogen) in 67 (75.3%) samples, but mismatched (positive pathogens were completely inconsistent) on the pathogen identification in 3 (3.4%) samples (**Figure 2B**). In comparison to CMT results (**Figure 2C**), the majority of the samples (85/99, 86%) were shown to be pathogen positive only in mNGS test. There were 11 samples that were double positive by two methods, with six samples partly matched in pathogens while five samples of pathogens mismatched.

Based on the clinical diagnosis of each sample, we next compared the diagnostic performance of mNGS, tNGS, and CMT. Among all 99 cases, 93 were clinically diagnosed as pulmonary infection and pathogens positive in analyzed BALF.

**Table 3** illustrates the performance characteristics of three methods. Owing to no FN samples detected by mNGS, the sensitivity was 100% for overall samples, the specificity was 50% because of an equal number of FP and TN samples (n=3), with an AUC of 0.75. The overall sensitivity for tNGS was 93.55%, significantly less than that for mNGS (p<0.05), and specificity was also 50%, with an AUC of 0.72. Although CMT achieved a 100% specificity because of no FP samples, its overall sensitivity was only 11.83%, with a poor AUC of 0.56. Specific per organism type, mNGS showed significantly better sensitivity for bacterial, fungal, and viral diagnosis than tNGS (p<0.05) and achieved a superior AUC of approximately 0.96 and 0.94 for bacteria and fungi, respectively. Particularly, for the diagnosis of *Pneumocystis jeroveci* (*P. jeroveci*) infection, both mNGS and tNGS performed well with AUC of 0.96 and 0.91, respectively.

**Table 3:** Performance characteristics of metagenomic and targeted NGS workflows and conventional microbiological test.

Performance category	No. of samples categorized as:				Sensitivity (%) (95% CI)	Specificity (%) (95% CI)	AUC (95% CI)
	TP	FP	TN	FN			
mNGS							
Overall	93	3	3	0	100 (96.11–100.0)	50 (11.81–88.19)	0.75 (0.4892–1.011)
Bacterial	48	1	47	3	94.12 (83.76–98.77)	97.92 (88.93–99.95)	0.96 (0.9157–1.005)
Fungal	47	3	46	3	94 (83.45–98.75)	93.88 (83.13–98.72)	0.94 (0.8848–0.9940)
Viral	64	24	11	0	100 (94.40–100.0)	31.43 (16.85–49.29)	0.66 (0.5360–0.7783)
*P. jirovecii*	31	3	64	1	96.88 (93.45–100.30)	95.52 (91.45–99.60)	0.96 (0.9167–1.007)
tNGS							
Overall	87	3	3	6	93.55 (86.48–97.60)	50 (11.81–88.19)	0.72 (0.4619–0.9736)
Bacterial	28	10	38	23	54.9 (40.34–68.87)	79.17 (65.01–89.53)	0.67 (0.5631–0.7776)
Fungal	38	8	41	12	76 (61.83–86.94)	83.67 (70.34–92.68)	0.8 (0.7067–0.8900)
Viral	54	16	19	10	84.38 (73.14–92.24)	54.29 (36.65–71.17)	0.69 (0.5787–0.8079)
*P. jirovecii*	28	4	63	4	87.5 (80.99–94.01)	94.03 (89.36–98.70)	0.91 (0.8330–0.9823)
CMT							
Overall	11	0	6	82	11.83 (6.055–20.18)	100 (54.07–100.0)	0.56 (0.3442–0.7740)
Bacterial	3	3	45	48	5.88 (1.25–10.52)	93.75 (88.98–98.52)	0.5 (0.3874–0.6162)
Fungal	4	1	48	46	8 (2.223–19.23)	97.96 (89.15–99.95)	0.53 (0.4157–0.6439)

TP, true positive; FP, false positive; TN, true negative; FN, false negative.

### 3.3 Comparison of detected pathogens and true positive rates

**Figure 3:**
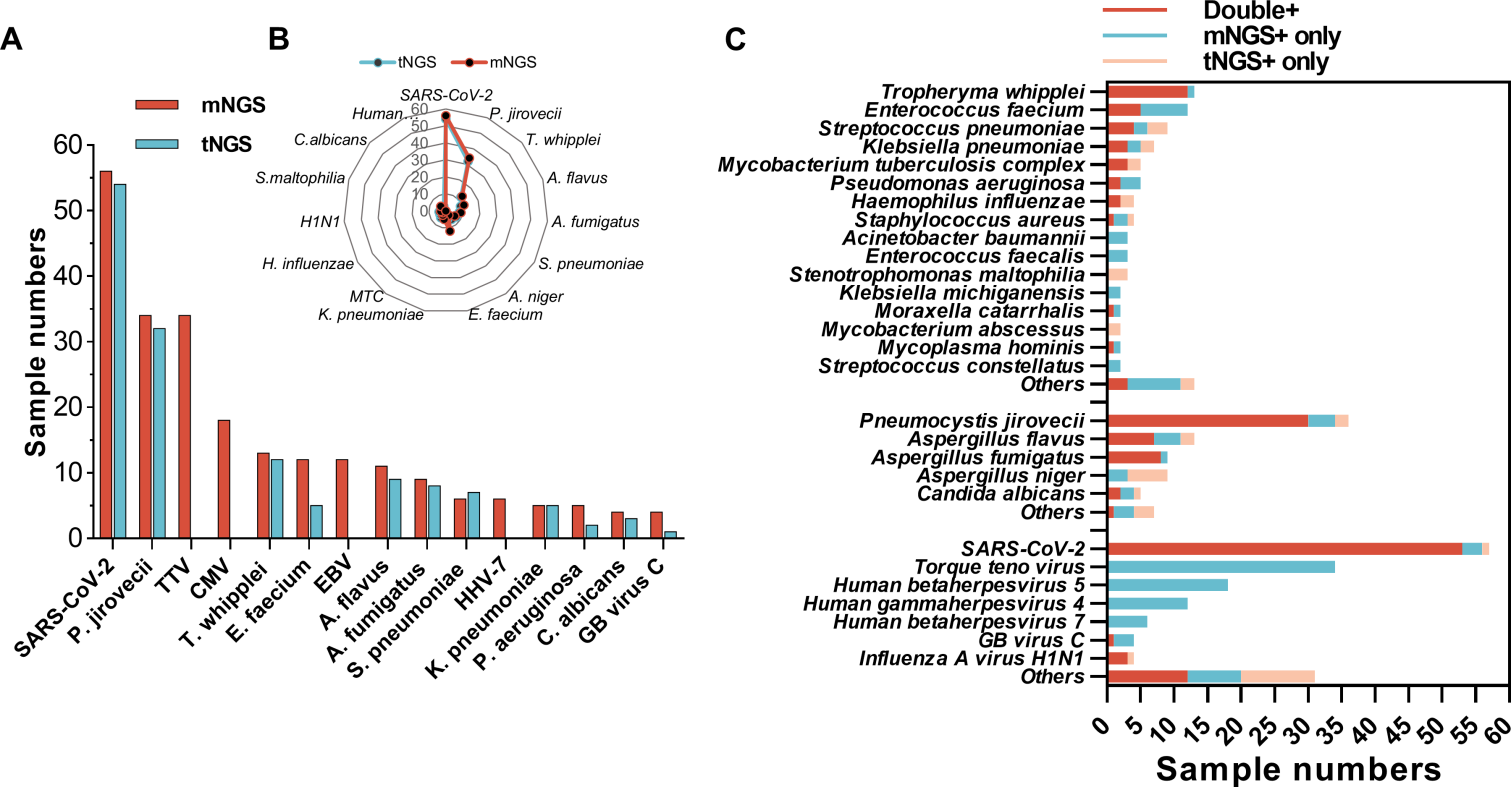
Comparison of detected pathogens spectrum between mNGS and tNGS. **(A)** Numbers of mNGS-detected top 15 microorganism identified by mNGS and tNGS. **(B)** Radar map shows the distribution of top 15 microorganism identified by tNGS and mNGS detected numbers. **(C)** Sample numbers of bacteria, fungi, and viruses identified by mNGS, tNGS, or both.

The top 15 microorganisms detected by mNGS and tNGS are shown in **Figures 3A, B**. SARS-CoV-2 and *P. jirovecii* were the top two microorganisms detected both by mNGS and tNGS, followed by Torque teno virus (TTV) and Human betaherpesvirus 5 (CMV) detected by mNGS besides the tNGS panel. The detected numbers of *Tropheryma whipplei*, *Enterococcus faecium*, *Aspergillus flavus*, *Aspergillus fumigatus*, *Streptococcus pneumoniae*, and *Klebsiella pneumoniae* were located at the 5th/3th, 6th/8th, 8th/4th, 9th/5th, 10th/6th, and 12th/9th, positions in mNGS and tNGS test, respectively. Human gammaherpesvirus 4 (EBV) and Human betaherpesvirus 7 (HHV-7) were detected in 12 and 6 samples by mNGS, but beyond the tNGS panel (**Figures 3A, B**).

Generally, a greater number of microbes was detected by mNGS than tNGS (**Figure 3**). Although most microbes within the tNGS panel could be co-detected by mNGS and tNGS in the same sample, there were some pathogens that were only being detected by mNGS, such as seven cases of *E. faecium*, three cases of *Pseudomonas aeruginosa*, and four cases of *P. jirovecii* and *A. fumigatus* (**Figure 3C**). Moreover, three numbers of *S. pneumoniae* and *Stenotrophomonas maltophilia*, six numbers of *Aspergillus niger*, and a few numbers of other microbes were only being detected by tNGS (**Figure 3C**).

**Figure 4:**
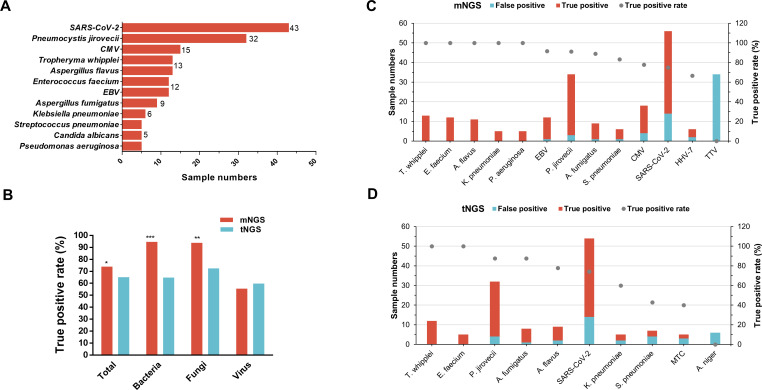
Comparison of detected pathogens true positive rate between mNGS and tNGS. **(A)** sample numbers of clinically recognized pathogens (only those with a sample size ≥5 are shown). **(B)** Comparison of the true positive rate of total microbes and microorganism type per between mNGS and tNGS. **(C, D)** The true positive and false positive numbers and true positive rate of corresponding microbes detected by the mNGS **(C)** and tNGS **(D)**, only microbes that identified in not less than five samples were shown.

According to diagnosed causative pathogens of each sample, all detected microbes by mNGS and tNGS were recognized as TP and false positive (FP). The causative pathogens with case numbers not less than five are shown in **Figure 4A**, indicating that the top 1 responsible pathogen was SARS-CoV-2 in 43 cases, followed by *P. jirovecii* in 32 cases, CMV in 15 cases, *T. whipplei* and *A. flavus* in 13 cases, *E. faecium* and EBV of 12 cases*, A. fumigatus* of nine cases, and *K. pneumoniae* of six cases. In addition, *S. pneumoniae*, *Candida albicans*, and *P. aeruginosa* infections were diagnosed in five samples. mNGS showed a total TP rate of 73.97% (216/292), significantly higher than that for tNGS (65.15%, 129/198, p<0.05) (**Figure 4B**). Specific for bacterial and fungal TP rates, mNGS also considerably outperformed tNGS (94.59% vs. 64.81% for bacteria, p<0.001; 93.85% vs. 72.58% for fungi, p<0.01). The TP rate between mNGS and tNGS for virus were comparable (55.56% for mNGS vs. 59.76% for tNGS, p=0.54).

In terms of TP rate for specific microbial species, 100% *T. whipplei* (13/13), *E. faecium* (12/12), *K. pneumoniae* (5/5), *P. aeruginosa* (5/5), and *A. flavus* (11/11) detected by mNGS were recognized as TP (**Figure 4C**). In addition, 11 of 12 (91.67%) EBV, 31 of 34 (91.18%) *P. jirovecii*, 8 of 9 (88.89%) *A. fumigatus*, 5 of 6 (83.33%) *S. pneumoniae*, 14 of 18 (77.78%) CMV, 42 of 56 (75%) SARS-CoV-2, 4 of 6 (66.67%) HHV-7, and none of 34 (0%) TTV that were detected by mNGS were TP (**Figure 4C**). As for tNGS (**Figure 4D**), *T. whipplei* (n=12) and *E. faecium* (n=5) also achieved a TP rate of 100%, followed by *P. jirovecii* (n=32) and *A. fumigatus* (n=8) with a TP rate of 87.5%. Furthermore, 7 of 9 (77.78%) *A. flavus*, 40 of 54 (74.07%) SARS-CoV-2, 3 of 5 (60%) *K. pneumoniae*, 3 of 7 (42.86%) *S. pneumoniae*, 2 of 5 (40%) *Mycobacterium tuberculosis* complex (MTC), and 0 of 6 (0%) *A. niger* identified by tNGS were TP (**Figure 4D**).

### 3.4 Distinctive clinical characteristics of TTV-positive versus TTV-negative recipients

**Figure 5:**
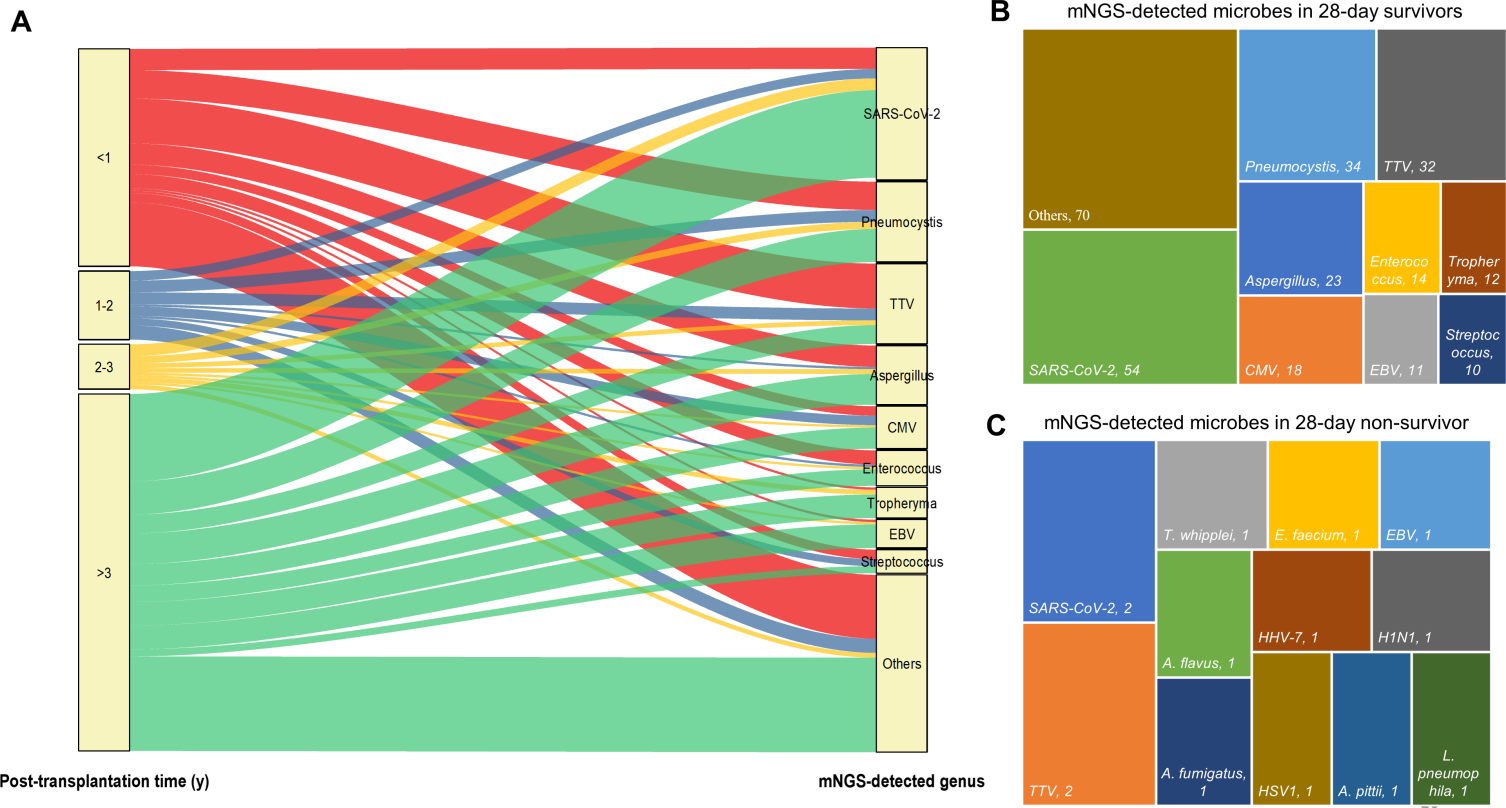
The relationship between mNGS-detected microbes and corresponding patients’ post-transplantation time and 28-day outcome. **(A)** Sankey diagram illustrates the relationship between mNGS-detected microbes at genus level and corresponding patients’ post-transplantation time. **(B, C)** mNGS detected microbes at genus level in 28-day survivors **(B)** and species in 28-day non-survivors **(C)**.

Regarding the identified microbes by mNGS and their corresponding post-transplantation time (**Figure 5A**), the majority of SARS-CoV-2 (37/56, 66.07%), *Aspergillus* (13/25, 52%), CMV (9/18, 50%), *Tropheryma* (10/13, 76.92%), and EBV (10/13, 83.33%) were detected from recipients who underwent surgery over 3 years as compared to the other three post-transplantation time periods. However, 55.88% (19/34) TTV were detected from recipients within 1-year post-transplant, followed by 23.53% (8/34) more than 3 years after surgery (**Figure 5A**). Most cases survived (96/99, 96.87%) at 28-day admission, and the top 5 detected genus by mNGS included SARS-CoV-2, *Pneumocystis*, TTV, *Aspergillus*, and CMV (**Figure 5B**). Among the three non-survived cases, two numbers of SARS-CoV-2 and TTV; one case of *T. whipplei*, *E. faecium*, EBV, *A. fumigatus*, and *A. flavus*; and five numbers of others were detected by mNGS (**Figure 5C**).

**Figure 6:**
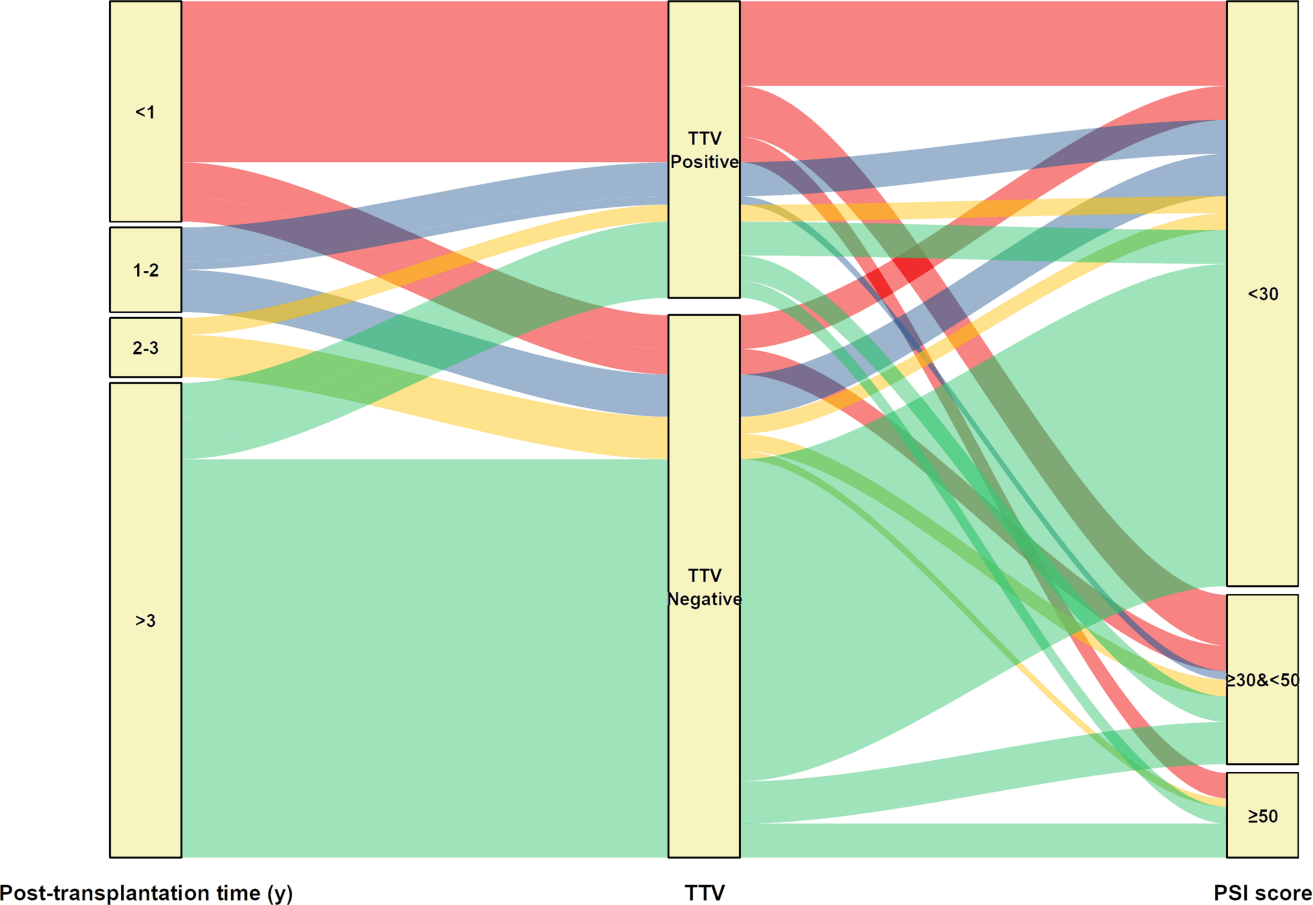
Sankey diagram illustrates the relationship between TTV positive or negative and corresponding patients’ post-transplantation time and PSI score.

We next compared the clinical characteristics between TTV-positive and TTV-negative patients by mNGS (**Table 3**). No significant difference was observed between sex, ages, and underlying diseases. Notably, we found that the constituent ratio of post-transplantation time between two groups largely differed (p<0.001), manifesting as that more than half (19/35, 54.29%) cases in the TTV-positive group were within 1 year after surgery, whereas most TTV-negative KTRs (47/64, 73.44%) were over 3 years post-transplantation (**Table 4**, **Figure 6**). In addition, TTV-positive cases had a higher ratio of eosinophils and basophilic granulocyte, along with increased D-dimer concentration and PSI score, when compared to TTV-negative patients (p<0.05) (**Table 4**, **Figure 6**).

**Table 4:** Comparison of clinical characteristics between TTV-positive and TTV-negative cases by mNGS.

Characteristics	TTV positive	TTV negative	p-value
Case number	35	64	/
Sex, n (%)	/		0.227
Male	21 (60)	46 (71.88)	/
Female	14 (40)	18 (28.13)	
Age, years (mean ± SD)	37.94 ± 9.12	40.67 ± 9.28	0.149
Underlying diseases	/		
Hypertension	25 (71.43)	50 (78.13)	0.457
Diabetes	1 (2.86)	3 (4.69)	0.658
Heart disease	2 (5.71)	1 (1.56)	0.249
Post-transplantation time	/	/	<0.0001
<1 year	19 (54.29)	7 (10.94)	/
1–2 years	5 (14.29)	5 (7.81)	
2–3 years	2 (5.71)	5 (7.81)	
>3 years	9 (25.71)	47 (73.44)	
Laboratory findings, median (Q1, Q3)	/		
WBC, 10^9^/L	6.53 (4.6, 10.75)	6.15 (4.67, 8.82)	0.553
Neutrophil count, 10^9^/L	4.96 (3.21, 8.91)	4.46 (3.44, 7.73)	0.647
Lymphocyte count, 10^9^/L	0.9 (0.5, 1.39)	0.67 (0.41, 1.07)	0.230
Eosinophils%	0.3 (0.04, 2.1)	0.1 (0, 0.28)	0.009
Basophilic granulocyte%	0.2 (0, 0.3)	0.1 (0, 0.1)	0.008
Platelet count, 10^9^/L	185 (125, 232)	160.5 (120.5, 216)	0.285
D-dimer, μg/ml	0.3 (0.12, 0.7)	0.16 (0.1, 0.3)	0.028
Severity and outcome	/		
PSI score	20 (10, 40)	10 (1, 20)	0.044
Total hospitalization time, days	19 (12, 31)	15.5 (10, 21.5)	0.094
28-day mortality, n (%)	2 (5.71)	1 (1.56)	0.285

## 4 Discussion

The application of NGS technologies for pathogens identification is a burgeoning field, and optimal practices are areas of active investigation. mNGS has been extensively served as a novel tool for defining potential causative microorganisms for infectious diseases including respiratory tract infections. Based on amplification of primers targeted specific pathogens, tNGS is economic but covers limited pathogens and has been recently applied for detecting respiratory tract pathogens (19, 32). In this study, mNGS significantly outperformed tNGS in identifying causative respiratory pathogens of BALF samples from KTRs. To our knowledge, this was the second study to compare mNGS and tNGS in respiratory infections, and the first was performed by Li et al. (19)., indicating that tNGS was comparable with mNGS in adults with pneumonia for pathogenic microorganism detection.

The immunological function of KTRs is clearly compromised by the long-term use of immunosuppressants, which raises the risk of postoperative infection. Consequently, KTRs are a population that is more susceptible to infection with SARS-CoV-2 and other infections (33). Among 99 KTRs of this study, 96 were diagnosed with pulmonary infection, and 43 of them were recognized as SARS-CoV-2 infection. This may be attributed to the sampling timing of our samples, which covered the partial COVID-19 pandemic period (Jan, 2022 to Sep, 2023). mNGS achieved a sensitivity of 100% for all samples owing to that the three non-infectious samples were also shown to be negative by mNGS. Meanwhile, because of the equal number of TN and FP samples (n=3), mNGS and tNGS showed a poor specificity of 50%. In previous studies, the sensitivity for BALF mNGS ranged from 55% to 100% (34–36), and specificity ranged from 60% to 93% (37, 38). Although the specificity of CMT was calculated to be 100% because of the zero FP samples, its sensitivity was only 11.83%, with a poor AUC of 0.56. Herein, the few numbers of diagnosed non-infectious samples may have influence on the superior sensitivity and underperforming specificity of mNGS and tNGS.

No significant difference in total, bacterial, and fungal positive rates between mNGS and tNGS was found, but a significant difference was observed in virus positive rate. This can be explained by the fact that the primary viruses including TTV, EBV, CMV, and HHV-7 detected by mNGS were beyond tNGS panel. As for pathogens that were covered by the tNGS panel, there were also microbes detected by mNGS or tNGS only in one sample. By sequencing nucleotides in patient samples, the mNGS approach, which surveys random samples of analyte DNA or RNA, might potentially identify all pathogens. But since host cells and nucleotides make up the majority of those samples (usually more than 90%), sequencing for microbial identification becomes far less effective (8, 39, 40). Although capture probes were employed for host DNA/RNA depletion in this study (8), host nucleic acid may also limit the overall analytical sensitivity of mNGS. Conversely, tNGS is a strategy that combines simultaneous quantitative analysis of amplified products with targeted primer extension, which can theoretically lower the amount of host nucleotides. On the contrary, we showed that mNGS exhibited better sensitivity than tNGS in both overall and per microorganism type infections. This was inconsistent with a recent report, which showed that the pathogen-targeted NGS had superior performance over mNGS for common causative pathogen detection in cerebrospinal fluid (CSF) for infectious meningitis/encephalitis (41). On the one hand, CSF is known to have high host background, with >200 cells per cubic milliliter typically observed for CSF cell counts (42), thus may hamper the sensitivity of mNGS. On the other hand, the amplification efficiency and threshold criteria for tNGS can largely impact the FP of reported pathogens, suggesting that tNGS techniques in our study still require refinement.

Followed by SARS-CoV-2, *P. jirovecii* was the second causative pathogen for pulmonary infection in this study. PJP is a severe and potentially fatal opportunistic illness that typically strikes recipients of solid organ transplants, especially in KTRs, who are vulnerable to respiratory infections (14, 43). However, diagnosis of PJP remains challenging due to its nonspecific clinical presentation and the inadequate performance of conventional diagnostic methods (44). Both for immunocompetent and immunocompromised cases, mNGS has been demonstrated to show superior diagnostic performance over CMT in PJP, with sensitivity ranging from 83% to 100% and specificity from 85% to 100% (45, 46). As observed in this research, both mNGS and tNGS exerted satisfying performance for *P. jirovecii*, manifested by sensitivity of 96.88% and 87.50%, specificity of 95.52% and 94.03%, AUC of 0.96 and 0.91, for mNGS and tNGS, respectively. No significant difference was observed in *P. jirovecii* detection between tNGS and mNGS, indicating that the potential value of tNGS for PJP diagnosis was comparable with mNGS.

Despite the fact that mNGS detected a large number of viruses in addition to SARS-CoV-2 in BALF samples, only a few of CMV and EBV were recognized as true positive and no TTV was identified as causative pathogen. This may lead to the relatively lower true positive rate for detected virus by mNGS than tNGS. Interestingly, we found that the proportion of TTV positivity decreased with the increase in post-transplantation time (73.08% for <1 year, 50% for 1–2 years, 28.57% for 2–3 years, and 16.67% for >3 years). TTV is a ubiquitous and non-pathogenic single-stranded DNA virus and has been proposed as a marker of functional immunity in immunocompromised patients (47). Increasing studies have reported the potential value of monitoring TTV after kidney transplantation for predicting events associated with excessive immune suppression and acute rejection (47–49). Similar to our findings, a previous study showed that TTV detection rate in the blood from KTRs reached peak point at 75 days after transplantation and subsequently dropped at 180 days and 360 days after transplantation (50). TTV viral load could gradually increase during the first 3 months post-transplantation in KTRs (51). The bidirectional movement of TTV from donor organ to recipient serum has been shown in lung transplant recipients by metagenomics analysis (52, 53). in addition, we did not observe statistical correlation between TTV-positive and post-transplant virus infection or rejection, but TTV positive cases were shown to have a higher ratio of eosinophils and basophil, along with increased D-dimer concentration and PSI score, than TTV-negative patients. Concentrations of eosinophils may be elevated by inflammatory conditions of the lower respiratory tract, including viral infection, and has been shown to be increased by TTV infection (54, 55). In this study, KTRs were administrated with reduced dosage of immunosuppressants 1-year post-transplantation. Therefore, the decreased positive rate for TTV as post-transplantation time increases may be explained by the application of immunosuppressors, which predispose KTRs to viral infections. Additionally, only one patient was shown to be TTV-3 positive in this study. Studies about the differences in the TTV genotypes among KTRs are scarce. Considering that the sequence data of mNGS could allow us to perform genomic analysis and phylogenetic tree construction, we intended to analyze the associations between TTV genotypes and clinical characteristic among KTRs in the subsequent study.

This study had certain shortcomings as well. First, the majority of patients (87.88%, 87/99) underwent treatment prior to tests, which may have impacted the performance of NGS and CMT, particularly for culture. Second, because our studied population were immunocompromised hosts, only three were diagnosed as pathogen negative among 99 KTRs, which may introduce bias into the estimates of sensitivity and specificity. Lastly, because this was a single-center, retrospective investigation, bigger sample sizes and prospective studies are required to validate our results.

Collectively, both mNGS and tNGS could provide valuable information in addition to CMTs. Although tNGS showed inferior performance over mNGS for respiratory causative pathogen detection in BALF for pulmonary infection among KTRs, tNGS also considerably outperformed CMT. NGS is expected to make larger contributions in the identification or exclusion of infections with additional workflow optimization.

## Data Availability

The datasets presented in this study can be found in online repositories. The names of the repository/repositories and accession number(s) can be found below: CRA016770 (GSA; https://ngdc.cncb.ac.cn/gsa).
